# Effects of Vitamin D Supplementation on Cognitive and Emotional Functioning in Young Adults – A Randomised Controlled Trial

**DOI:** 10.1371/journal.pone.0025966

**Published:** 2011-11-04

**Authors:** Angela J. Dean, Mark A. Bellgrove, Teresa Hall, Wei Ming Jonathan Phan, Darryl W. Eyles, David Kvaskoff, John J. McGrath

**Affiliations:** 1 Queensland Brain Institute, The University of Queensland, St Lucia, Australia; 2 Queensland Centre for Mental Health Research, The Park Centre for Mental Health, Richlands, Australia; 3 Department of Psychiatry, School of Medicine, The University of Queensland, St Lucia, Australia; Chiba University Center for Forensic Mental Health, Japan

## Abstract

**Background:**

Epidemiological research links vitamin D status to various brain-related outcomes. However, few trials examine whether supplementation can improve such outcomes and none have examined effects on cognition. This study examined whether Vitamin D supplementation led to improvements in diverse measures of cognitive and emotional functioning, and hypothesised that supplementation would lead to improvements in these outcomes compared to placebo.

**Methods/Principal Findings:**

Healthy young adults were recruited to a parallel-arm, double-blind trial conducted at The University of Queensland. Participants were randomly allocated to receive Vitamin D (one capsule daily, containing 5000 IU cholecalciferol) or identical placebo capsule for six weeks. All participants and outcome assessors were blinded to group assignment. Primary outcome measures assessed at baseline and 6 weeks were working memory, response inhibition and cognitive flexibility. Secondary outcomes were: hallucination-proneness, psychotic-like experiences, and ratings of depression, anxiety and anger. 128 participants were recruited, randomised and included in primary analyses (vitamin D n = 63; placebo n = 65). Despite significant increases in vitamin D status in the active group, no significant changes were observed in working memory (F = 1.09; *p* = 0.30), response inhibition (F = 0.82; p = 0.37), cognitive flexibility (F = 1.37; *p* = 0.24) or secondary outcomes. No serious adverse effects were reported.

**Conclusions:**

Our findings indicate that vitamin D supplementation does not influence cognitive or emotional functioning in healthy young adults. Future controlled trials in targeted populations of interest are required to determine whether supplementation can improve functioning in these domains.

Australian and New Zealand Clinical Trials Registry; ACTRN12610000318088.

## Introduction

Vitamin D is a nuclear steroid hormone with diverse physiological roles. In addition to its well-established role in calcium homeostasis, vitamin D is being reconsidered as a neuroactive steroid [1], [2], [3]: the distribution of the vitamin D receptor in the human brain has been characterised [4]; the enzyme responsible for synthesis of the active form of vitamin D is present in the brain [5]; and animal research indicates that vitamin D is important for brain development [1].

Epidemiological studies link vitamin D status to a range of brain-related outcomes. Low concentrations of vitamin D have been associated with impairments in cognitive functions such as memory and orientation [6], [7], [8], executive function impairments [9], [10], and diagnosis of dementia and Alzheimer's disease [11]. Increased dietary intake of vitamin D has been associated with lower rates of self-reported psychotic-like experiences [12], and vitamin D status at birth or in early life is associated with reduced risk of schizophrenia in later life [13], [14]. Many studies report an association between low vitamin D status and depressive symptoms [15], [16], [17].

In response to such findings, The Institute of Medicine [18] has called for well-conducted, randomised controlled trials to examine whether vitamin D supplementation can improve brain-related outcomes. A number of trials have assessed the effects of vitamin D on depression, but results have been conflicting [19], [20]. No trials were identified that examined the effect of vitamin D supplementation on cognition or psychotic-like experiences. To address this gap, we conducted a randomised controlled trial to assess whether vitamin D supplementation would lead to improvement in (i) key cognitive functions of working memory, response inhibition and cognitive flexibility; (ii) psychotic-like experiences and hallucination proneness; and (iii) key emotional states of depression, anxiety and anger.

## Methods

### Participants

Participants were healthy volunteers recruited through advertising at the University of Queensland. Inclusion criteria were at least 18 years of age, with sufficient English language skills required to complete the study protocol. Individuals were excluded if they met any of the following criteria: current use of vitamin D or calcium supplements; history of adverse reactions to vitamin supplements; current or past diagnosis of a mood or psychotic disorders; history of neurologic illnesses including cerebrovascular accident, CNS tumours, head trauma, multiple sclerosis, epilepsy, movement disorders or migraine treatment; current or recent (1 year) history of dependence on alcohol or illicit substances; intellectual disability; pregnancy or current breast feeding, or potential to become pregnant during the trial; history of severe renal impairment. After telephone screening, potentially eligible participants were invited to attend the research clinic for assessment. After they had been provided with verbal and written information about the study (including the patient information sheet), eligible participants provided written informed consent. No changes to eligibility criteria or other methods were made after trial commencement. The trial was approved by the University of Queensland Medical Research Ethics Committee, and pre-registered with the Australian and New Zealand Clinical Trials Registry (ACTRN12610000318088). The study was funded by Queensland Health. The protocol for this trial and supporting CONSORT checklist are available as supporting information; see Checklist S1 and Protocol S1.

### Trial design

This study was a randomised controlled, parallel-arms trial, comparing vitamin D supplementation to placebo (1∶1 allocation ratio).

### Interventions

Participants were randomly assigned to receive either Vitamin D (5000IU cholecalciferol) or placebo. These were provided as identical microcellulose capsules, prepared by an external clinical trials service. Placebo capsules contained lactose. Participants were provided with 6 weeks of study medication, and instructed to take one capsule daily. To optimise adherence, participants were sent weekly reminders via email or text message.

### Implementation of randomisation and blinding

Randomisation sequence was generated by the external clinical trials site. To ensure that each treatment group was uniformly represented over the time course of the study, a varying-block randomisation protocol was used (randomly determined block sizes of 4 or 6). After provision of written consent, each participant was assigned to the next consecutive participant number by two researchers not involved in the generating the randomisation sequence. All investigators, outcome assessors and participants were blinded to treatment allocation procedures and treatment group throughout the study. After completion of the final assessment, participants were asked to guess whether they had received vitamin D or placebo based on their overall subjective impression of change during the study.

### Outcome assessments

The following assessments were conducted at baseline and after 6 weeks of vitamin D supplementation. The cognitive measures were selected based on their limited capacity to generate ceiling effects in healthy populations.

#### Working memory – N-back

The N-Back task is a widely used computer-based test of visuospatial working memory [21]. Participants were presented with a screen containing a scattered arrangement of ten squares: every 500 ms, a different square would become shaded. Participants were required to identify whether the position of the shaded square was the same as that presented three screens previously (3-back). After a demonstration and practice block to ensure participants understood the task, four blocks were administered, each containing 50 trials. The dependent measure used was the proportion of correct responses.

#### Response inhibition - Stop signal task

Response inhibition is a specific executive function which involves suppression of behavioural impulses and is measured using tasks such as the Stop Signal Task [22]. The stop-signal task requires the cancellation of a prepotent ‘go’ response upon presentation of an infrequent ‘stop’ signal. Stop-signal inhibition can be viewed as a race between two competing ‘go’ and ‘stop’ processes. By introducing a delay between the presentation of the go and any subsequent stop signal, one can bias the outcome of the race. The stop-signal reaction time (SSRT) was derived as the mean reaction time to go-stimuli minus the stop signal delay for the 50% inhibition threshold [22]. A lower (i.e. faster) SSRT is indicative of better inhibitory control. In this study, there was a 64-trial practice block, followed by four 128-trial blocks with a break scheduled in between each block.

#### Cognitive flexibility - Set shifting task

This is a computer-based measure that requires alternating between responses to different categories of stimuli [23]. Participants were presented with coloured shapes and required to identify either the colour or shape depending on an alternating response cue. This task comprised four blocks of 48 trials. The dependent measure was the switch-cost reaction time, calculated as difference in reaction time between shift trials (where the response cue was the opposite of the previous trial) and non-shift trials (where the response cue was the same as the previous trial). A lower switch-cost represents better cognitive flexibility.

#### Psychotic-like experiences

The Peters Delusion Inventory-21 (PDI-21) is a 21-item self-report measure of delusional-like experiences [24]. Items were statements of experiences that illustrate misattribution of emotions in everyday situations. One such item was ‘Do you ever feel as if things in magazines or on TV were written especially for you?’ Participants received a total score out of 21, with higher scores indicating greater proneness to delusional-like experiences.

#### Hallucinatory proneness

The white noise task involves presenting participants with one of three types of auditory stimuli via headphones: (a) white noise only; (b) white noise and audible speech of neutral content; and (c) white noise and barely audible speech of neutral content. Twenty-five fragments of each group were presented in random order. Participants were asked to indicate whether they had heard a voice or not. The primary outcome for analysis was the number of times participants indicated hearing a voice in the white noise only condition. This measure of speech illusion is thought to indicate proneness to psychosis, and higher scores have been associated with schizotypy compared to controls [25].

#### Depressive symptoms

The Beck Depression Inventory (BDI) is a 21-item self-report questionnaire with item scores ranging from 0 to 3 and a total score ranging from 0 to 63. It has been verified as a reliable and valid screening instrument to detect intensity of depression in a variety of populations and has also been employed to measure treatment response when used in a pre- and post-test design [26].

#### State Anxiety

The State-Trait Anxiety Inventory (STAI) is a 40-item self-report questionnaire used to measure both current anxiety (state) (20 items) and anxiety as a more enduring stable personality characteristic (trait) (20 items). In this study we utilised the State Anxiety subscale [27].

#### State Anger

The State-Trait Anger Expression Inventory (STAXI-2) is a 57-item self report questionnaire which measures the experience, expression and control of anger. In this study, the key outcome was the State Anger subscale [28].

#### Adverse effects

The Treatment Emergent Symptom Scale is a 31-item self-report questionnaire widely used in psychopharmacology trials [29]. A total score was utilised to assess a range of potential adverse effects. Participants were also invited to spontaneously report any other potential symptoms occurring during the study.

### Quantification of vitamin D status

Blood samples were collected as finger prick capillary whole blood spots. These were collected at baseline and at the 6-week follow-up using a single-use, automated lancet device. Vitamin D status is internationally quantified as 25-hydroxyvitamin D3 (25OHD3). 25OHD was extracted from the dried blood spots, and assayed using a highly sensitive tandem mass spectroscopy assay optimised for these samples [30]. Results are reported as sera concentrations after correction for peripheral haematocrit [30].

### Sample size calculation

For the primary two-group comparisons, a sample size of approximately 128 will have a power of 80% to detect a small-medium effect size (equivalent to *d* = 0.4). This is based on two occasions of response, a moderate within-subject correlation of 0.5, and a two-sided significance level of 5%.

### Statistical methods

Treatment outcomes were analysed using mixed-effects modelling. A mixed effects model was created for each outcome variable. Primary analysis examined the effect of treatment allocation group (vitamin D versus placebo) on treatment outcome, with time and treatment group entered as fixed effects. The adequacy of each model was assessed by examining residuals for heterogeneity and normality. Primary analysis included all randomised participants (intention to treat analysis). Two secondary analyses were conducted:

- The effect of change in vitamin D concentrations on treatment outcome. Two groups were determined based on the mean change in concentrations of 25OHD3; change in vitamin D group and time were entered as fixed factors in mixed models.
- The effect of change in vitamin D concentrations on treatment outcome, in the subset of individuals who had lower serum concentrations of 25OHD3 at baseline. Vitamin D deficiency is often defined by serum concentrations of 25OHD3 less than 50 nmol/L. However, results indicated that too few participants met this criterion. As such, for this analysis, participants were classified *post hoc* as having low baseline serum 25OHD3 concentrations if they scored below the median value. Within this subgroup, secondary analysis (i) was repeated.

## Results

### Recruitment and participant flow

**Figure 1:**
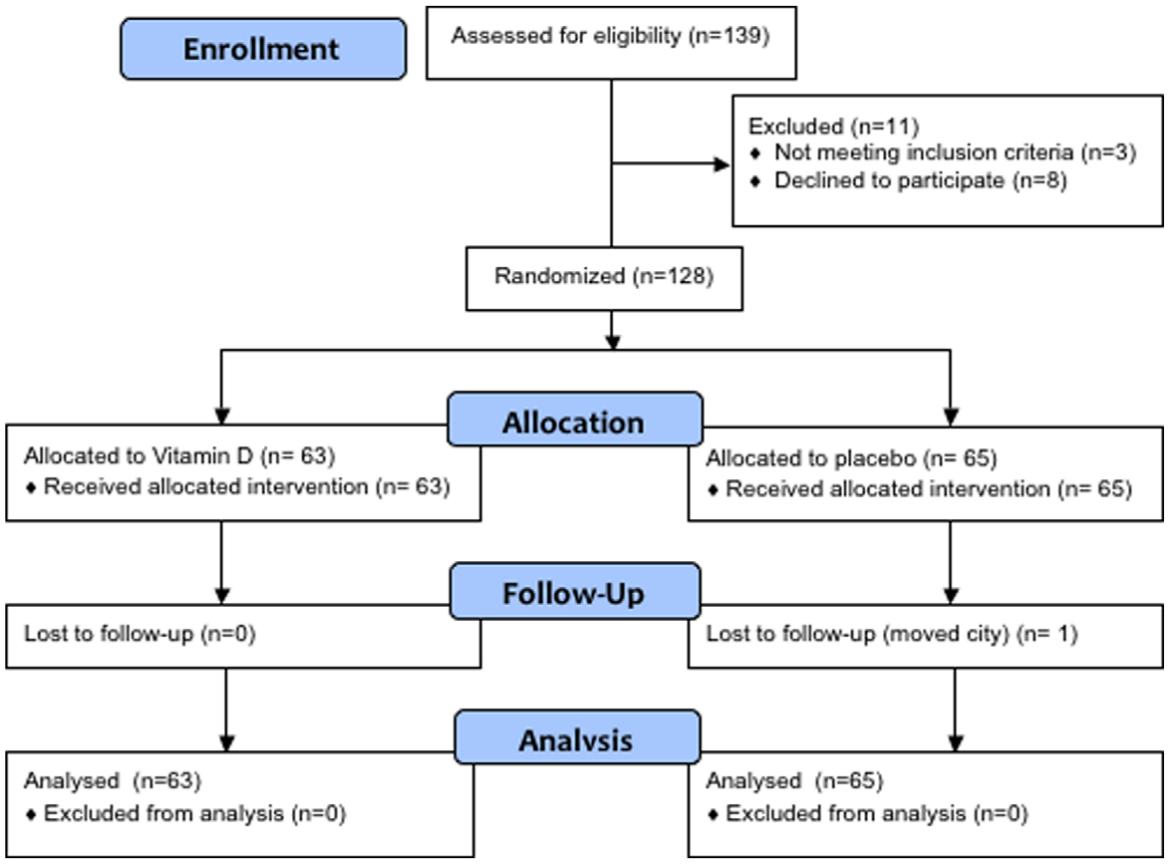
Flow chart of trial participation.

Trial recruitment began in February 2010 and was follow-up was completed in September 2010 after successful recruitment of the target sample size. One hundred and thirty nine individuals contacted the recruitment team and were screened for eligibility. Eight individuals declined to participate (not available for the follow-up session), and three were excluded (one had an acquired brain injury and two were taking psychotropic medication). This left 128 eligible participants who consented to participate. Sixty-three were randomly allocated to vitamin D capsules, and 65 were allocated to placebo capsules. Only one participant was lost to follow-up. All participants were included in analysis of primary outcomes. Participant screening and flow is described in Fig. 1.

### Participant characteristics at baseline

The mean age of participants was 21.8 years (s.d. = 2.9; Range 18–30), and more than half were female (57%; 73/128). Table 1 describes participant characteristics at baseline for all key demographic and outcome variables. Participants allocated to placebo exhibited higher scores on the PDI compared to those allocated to vitamin D (t = 2.02; p<0.05). No other differences were observed between participant groups.

**Table 1:** Comparison of baseline characteristics between participants allocated to Vitamin D and placebo (continuous variables are expressed as mean (standard deviation); categorical variables are expressed as a proportion).

	Vitamin D	Placebo	Statistic
	(n = 63)	(n = 65)	
Age (years)	21.45 (2.96)	22.06 (2.74)	t = 1.21
Gender (% female)	61.9% (39/63)	52.3% (34/65)	χ^2^ = 1.20
Ethnicity	European 41.9% (26/62)	European 33.8% (22/65)	χ^2^ = 1.33
	Asian 45.2% (28/62)	Asian 55.4% (36/65)	
	Other 12.9% (8/62)	Other 10.8% (7/65)	
Outdoor time (hours/week)	16.95 (4.19)	16.46 (5.44)	t = −0.57
25OHD3 (nmol/L)	76.25 (19.63)	77.23 (20.95)	t = 0.26
Working memory (Correct hits)	0.57 (0.18)	0.55 (0.20)	t = −0.61
Response inhibition (SSRT)	210.42 (40.77)	207.15 (39.22)	t = −0.45
Cognitive flexibility (ms)	246.34 (166.29)	256.93 (185.25)	t = 0.34
PDI total score	6.11 (3.05)	7.45 (4.33)	t = 2.02*
White noise task (count)	0.29 (0.73)	0.31 (0.81)	t = 0.20
BDI	7.24 (7.82)	5.72 (5.56)	t = −1.27
State anxiety	36.29 (10.10)	34.15 (8.31)	t = −1.31
State anger	16.41 (3.95)	16.26 (3.76)	t = −.22

*p<0.05.

### Vitamin D status

In all participants, mean concentrations of 25OHD3 at baseline were 76.6 nmol/L (SD 19.9; Range 41.1–149.3 nmol/L; median 75.0 nmol/L). Only ten participants had baseline concentrations lower than 50 nmol/L, the cut-off typically used for insufficiency [31].

There was a significant difference between treatment groups with regard to changes in vitamin D status over time (F = 21.44; p<0.001; *d* = 0.83). Participants allocated to vitamin D exhibited an increase over time (from a mean of 76.2 nmol/L at baseline to 98.0 nmol/L) whereas those allocated to placebo did not (baseline 77.20 nmol/L; follow-up 75.37 nmol/L) (Table 2).

**Table 2:** Pre and post data for vitamin D status and all outcome measures for primary analysis comparing outcomes between participants allocated to Vitamin D and those allocated to placebo (All randomised participants were included in all analyses: Vitamin D n = 63; placebo n = 65).

		Vitamin D		Placebo				
		Mean (SE)	95% CI (mean)	Mean (SE)	95% CI (mean)	F	*p*	*d*
25OHD3 (nmol/L)	Baseline	76.2 (2.6)	71.0–81.4	77.2 (2.6)	72.0–82.4	21.44	<0.001	0.83
	Follow-up	98.0 (3.3)	91.4–104.6	75.4 (3.3)	68.9–81.9			
Working memory	Baseline	0.57 (0.02)	0.52–0.62	0.55 (0.02)	0.50–0.59	1.09	0.30	0.19
(N Back - Correct hits)	Follow-up	0.62 (0.02)	0.57–0.66	0.62 (0.02)	0.58–0.67			
Response inhibition	Baseline	211.13 (5.18)	200.87–221.38	208.83 (5.08)	198.77–218.88	0.82	0.37	0.16
(Stop Signal SSRT, ms)	Follow-up	195.71 (4.67)	186.45–204.96	198.85 (4.59)	189.78–207.93			
Cognitive flexibility	Baseline	246.34 (22.18)	202.44–290.24	255.59 (21.96)	212.12–299.05	1.37	0.24	0.21
(Switch cost RT, ms)	Follow-up	143.91 (17.15)	109.98–177.84	185.75 (16.98)	152.16–219.34			
White noise task	Baseline	0.29 (0.10)	0.09–0.48	0.31 (0.10)	0.12–0.50	0.02	0.88	0.03
(Hallucination count)	Follow-up	0.37 (0.16)	0.05–0.68	0.35 (0.16)	0.04–0.67			
PDI (total)	Baseline	6.11 (0.48)	5.16–7.06	7.45 (0.47)	6.51–8.38	1.01	0.32	0.18
	Follow-up	5.58 (0.48)	4.62–6.53	6.49 (0.48)	5.56–7.43			
BDI (total)	Baseline	7.24 (0.84)	5.58–8.90	5.72 (0.83)	4.09–7.36	0.44	0.51	0.12
	Follow-up	6.40 (0.85)	4.73–8.07	5.38 (0.83)	3.74–7.02			
State anxiety	Baseline	36.29 (1.20)	33.91–38.66	34.15 (1.19)	31.81–36.50	1.44	0.23	0.21
	Follow-up	36.68 (1.21)	34.28–39.08	36.08 (1.19)	33.73–38.42			
State anger	Baseline	16.41 (0.49)	15.45–17.37	16.26 (0.48)	15.32–17.21	1.30	0.26	0.20
	Follow-up	15.81 (0.33)	15.15–16.47	16.43 (0.32)	15.79–17.07			

The following cut-offs were used for secondary analysis: (i) the mean change in 25OHD3 concentrations was 10.04 nmol/L and outcomes were compared between those scoring above this and those scoring below; (ii) the median baseline concentration of 25OHD3 was 75.0 nmol/L and the effects of supplementation was examined in the sub-group of participants scoring below this value (n = 59).

### Outcomes

Primary analysis demonstrated that vitamin D supplementation was associated with no change on any of the outcome measures. For example, working memory (as measured by number of correct hits on the N-back task) exhibited an improvement over time in all participants (F = 16.31; p<0.001) but no significant difference between groups over time (F = 1.09; p = 0.30). Similar results were observed with all other measures, with no significant differences over time between participants receiving vitamin D and those receiving placebo for response inhibition (F = 0.82; p = 0.37), cognitive flexibility (F = 1.37; p = 0.24), hallucination proneness (F = 0.02; p = 0.88), delusions (F = 1.01; p = 0.32), depressive symptoms (F = 0.44; p = 0.51), state anxiety (F = 1.44; p = 0.23), and state anger (F = 1.30; p = 0.26). Results for all treatment × time analyses are provided in Table 2.

Secondary analysis examining the effect of change in vitamin D concentrations on treatment outcome in (a) all participants, and (b) participants with low serum concentrations of 25OHD3 at baseline (<75.00 nmol/L) reported similar findings. No differences between participants exhibiting higher than average change in 25OHD3 concentrations over time and remaining participants were observed on any outcome measure (data not shown).

Vitamin D was well tolerated. There was one report of transient rectal bleeding from one participant receiving placebo. No other adverse effects were reported during the trial. There were no differences between vitamin D and placebo groups on total score on the Treatment Emergent Symptom Scale (t = −1.13; p = 0.26). There was no difference in participant perceptions of their treatment allocation, with the majority believing they received placebo: 73.8% (45/61) of participants receiving vitamin D and 76.6% (49/64) of those receiving placebo believed they received placebo (χ^2^ = 0.13; p = 0.84).

## Discussion

This trial was the first study, to our knowledge, specifically designed to assess the effects of vitamin D supplementation on key measures of cognitive and emotional functioning. Our findings indicate that vitamin D supplementation or increasing serum concentrations of 25OHD3 had no beneficial effects on (i) core executive functions of working memory, response inhibition or cognitive flexibility, (ii) psychotic-like experiences and hallucination proneness, or (iii) ratings of depression, anxiety or anger. This trial utilised an adequate dose of vitamin D, was placebo-controlled, was adequately powered for primary analyses and displayed high retention rates. As such, our findings appear to represent a true non-effect of vitamin D supplementation on cognitive and emotional functioning in healthy young adults.

These findings should be viewed in the context of an increasing number of studies associating low vitamin D status with impairments in mood and cognitive function and subsequent recommendations of widespread supplementation [32], [33]. The Institute of Medicine issued a recent report on vitamin D [18]. This report drew attention to the fact that biological claims linking vitamin D status to brain-related outcomes have some biological plausibility, but observational studies related to these outcomes have been difficult to interpret due to residual confounding and/or reverse causality. In particular, individuals with depression and impaired cognition are prone to reduced outdoor activity, which in turn could lead to reduced vitamin D status. Even if low serum vitamin D was found to be causally contributing to cognitive and emotional impairments, it cannot be assumed that supplementation will ameliorate the impairment. The Institute of Medicine called for well-conducted, randomised controlled trials to examine whether changing vitamin D status can improve outcomes.

Our cognition findings support other studies which have examined the relationship between vitamin D status and cognitive functioning in young adults. For example, the NHANES study [31] reported no association between vitamin D status and neurocognitive functioning in adolescents and adults. Fewer clinical studies have examined the relationship between vitamin D status and psychotic-like experiences. Our findings indicate that vitamin D supplementation is unlikely to influence psychotic-like experiences or delusion ratings in healthy volunteers. The relationship between vitamin D supplementation and mood is more complex. A number of controlled trials report no effect of vitamin D on mood [20], [34]. Two double-blind controlled trials do report some positive effects of vitamin D supplementation on mood [19], [35]. However, these studies have a number of limitations, such as using analyses that do not incorporate the effects of both treatment group and time [35], or reporting only minor reductions on depression measures that are unlikely to be clinically significant [19]. As such, no well-conducted trials to date suggest that vitamin D supplementation is associated with clinically significant changes in mood. No previous studies have examined the relationship between vitamin D and other emotional states such as anger and anxiety.

### Limitations

Our study has a number of limitations. Our sample consisted of healthy young adults, who were free of major psychiatric illness and cognitive impairment. As such, our findings may not generalise to clinical populations exhibiting cognitive impairment or emotional disorders. Further controlled trials need to be conducted in key populations of interest, including those with established deficiency. Existing studies have not identified a putative ‘effective dose’ or ‘necessary threshold’ for vitamin D to improve brain function. The dose used in this study was higher than that used in many other studies, and thus, our negative findings are unlikely to have resulted from inadequate doses. The putative mechanism of action of vitamin D on the adult brain is not established. It is unclear whether vitamin D is active only in individuals who are deficient, or whether it also exerts specific pharmacological effects in those with adequate concentrations of vitamin D. Whilst our sample was adequately powered for primary analyses and the first stage of secondary analyses, it is possible that too few participants exhibited low baseline concentrations of 25OHD3 to conduct well-powered analysis in this potentially important subgroup. Analyses were not adjusted for multiple outcome analyses; however, this strengthens the likelihood that our negative findings represent a true lack of effect in this group. It is also feasible that low vitamin D status operates over many years, and that brain-related outcomes are ‘long latency’ disorders [36]. Just as hip fracture and osteoporosis only emerge as adverse health outcomes associated with decades of vitamin D insufficiency, perhaps a similar latency is required for brain outcomes. There is robust evidence from in vitro and animal studies indicating that the active form of vitamin D has neuroprotective properties [3]. Thus, it is possible that chronic hypovitaminosis D could leave individuals more vulnerable to subsequent neurobiological insults.

In conclusion, our findings indicate that vitamin D supplementation does not influence cognitive or emotional functioning in healthy young adults. Despite promising clues from observational studies, there are currently no clinical data that supports the use of vitamin D supplementation as a treatment for cognitive or emotional impairments. Although detection and treatment of vitamin D insufficiency remains important for a range of health outcomes (e.g. bone health), future controlled trials in targeted populations of interest are required to elucidate the causal contribution of vitamin D status to brain-related outcomes and determine whether supplementation can improve functioning in these domains.

## Supporting Information

Checklist S1 **CONSORT Checklist.**
(DOC)

Protocol S1 **Trial Protocol.**
(PDF)
