# Supplementary material for: Effects of Vitamin D Supplementation on Cognitive and Emotional Functioning in Young Adults – A Randomised Controlled Trial
Source: PLoS One. 2011 Nov 4;6(11):e25966. doi: 10.1371/journal.pone.0025966 (PMC3208539; doi:10.1371/journal.pone.0025966)
Supplement: Protocol S1 — Trial Protocol. (PDF) [file pone.0025966.s002.pdf]

screening, will be randomly allocated to receive either (i) daily vitamin D capsules OR (ii) identical looking placebo capsules. At the start and end of the study we will assess various component of brain functioning, including attention, executive function, response inhibition, and general mood and emotions. We anticipate that this study will provide us with important information about the effects of enhancing vitamin D blood concentrations on brain function in healthy adults.

5) Give details of the research plan:

Note: The committee needs sufficient information to put into context the ethical considerations listed in later questions.

Note: This section should be completed in LAY LANGUAGE*as much as possible* so that it can be understood and appreciated by all Committee Members, including Lay Members.

Note: For application to the MREC – please keep response to a MAXIMUM of 2 pages.

METHODS

Subjects

We will aim to recruit subjects from students and staff attending the University of Queensland, aged 18-55 years.

Participant Inclusion Criteria: This study will recruit healthy controls. Inclusion criteria include: aged 18 years or over, and provision of consent to participate.

Exclusion criteria: Subjects will be excluded if they have any of the following: 1) current use of vitamin D or calcium supplements; 2) having past adverse reactions to vitamin supplements; 3) current or recent use of psychotropic medications or medications which may influence vitamin D absorption (e.g. cholestyramine, orlistat) 4) current or past diagnosis of a mood or psychotic disorders; 5) neurologic illnesses including CVA, CNS tumours, head trauma, multiple sclerosis, epilepsy, movement disorders or migraine in treatment; 3) drug dependency on alcohol or illicit substances; 6) intellectual disability 7) pregnancy or current breast feeding, or potential to become pregnant during the trial; 8) history of severe renal impairment, 9) recent or current use of immunosuppressive therapy.

Gender ratio: both males and females will be recruited for this study (no specific ratio required).

Study duration: 6 weeks

General procedure

Potential participants will be screened for eligibility (see below). Those eligible will be offered the opportunity to participate and informed consent will be conducted. Participants will then be randomly allocated to one of two treatment groups and receive study medication (placebo or 5000 IU Vitamin D daily) for a period of 6 weeks. Major assessments will be conducted at baseline and 6 weeks; brief follow-up assessments will take place at every two weeks.

Group allocation & blinding

Participants will be randomly allocated to either placebo or 5000 IU vitamin D. Blinding of treatment allocation will be assured by the separation of randomisation services and researchers involved in assessment. To ensure that each treatment group is uniformly represented over the time course of the study, a varying-block randomisation protocol will be used: varying block sizes of 4 and 6 (where there are even numbers of each treatment group represented every cycle of 4 or 6 participants) will be used to ensure that randomisation outcome cannot be predicted. Each new participant will be assigned to the next consecutive patient number. All involved in the project (investigators, outcome assessors and participants) will be blinded to treatment group.

Treatment groups and dosing schedule

- Vitamin D (Cholecalciferol) 5000 IU capsules, one per day.
- Placebo capsules: identical looking placebo capsules, one per day.

All participants will be instructed to take one capsule per day. These can be taken with or without food at any time of the day. Preparation of study medications (vitamin D and placebo capsules) will be prepared and dispensed by Wesley Hospital Pharmacy.

Assessments

Baseline assessment will include the following instruments:

- General demographic screening tool
- Health Screening Questionnaire, will assess health issues, medical history and medication use.
- ADHD Screening Questionaire

- National Adult Reading Tasks (NART)

Follow-up assessments will be conducted at baseline and 6-week follow-up.

- Neurocognitive function: comprehensive neurocognitive assessment includes measures of inhibitory control (stop-signal reaction time), sustained attention (Sustained Attention to Response Test), spatial working memory (N-Back), white noise task, and cognitive flexibility (set shifting task)
- Emotional function: Beck Depression Inventory; State Trait Anxiety Inventory; State Trait Anger Inventory; Peters' Delusional Inventory; The Aggression Questionnaire.
- Sun, Skin and Diet Questionnaire (SSDQ) is a self-report measure of recent sun exposure, time spent outdoors, use of sun protection, dietary intake vitamin D, and use of vitamin D supplements.
- Treatment Emergent Symptom Scale will be used to screen for potential side effects at each follow-up.
- Medication adherence will be assessed using self report and pill counts.

Biological testing - assessment of vitamin D status
Blood samples will be collected at baseline and 6 week follow-up to determine serum concentrations of 25-hydroxy vitamin D. To avoid the need for venipuncture, two drops of blood will be collected using an automated lancet device from a finger prick (as used in standard diabetes blood glucose monitoring). These devices are safe and cause minimal discomfort (e.g. comparable to a paper cut). The research team participating in collection of blood samples will be trained in appropriate procedures (include universal blood precautions and use of the device) by Professor John McGrath (a registered medical practitioner and psychiatrist). Blood will be blotted onto standard filter paper, for later testing using a highly sensitive tandem mass spectroscopy system (Eyles et al., 2009) in the laboratory of Dr Darryl Eyles.

Biological testing – genetic screening
Participants will be also asked to provide a salivary sample, to enable DNA extraction for genetic testing. Genetic testing will examine genes involved in neurocognitive function such as the dopamine transporter (DAT).

Sample size and time frame
The estimated time frame of for this study is to complete within 3 years. This incorporates a recruitment period of up to 2 years.

Sample size calculations were based on the primary outcome measure (Response inhibition – Stop signal task, with SSRTs treated as a continuous variable). Because there are no human data to guide calculation of expected effect sizes, power calculations were based on a small effect size. Additionally, powering studies to detect small effect sizes permits controlling for sample heterogeneity. For the primary two-group comparisons, a sample size of between 126-132 will have a power of 80% to detect a small effect size. This is based on two response occasions, a moderate within-subject correlation of 0.5, and a two-sided significance level of 5%.

Statistical analysis
Changes over time in the sample as a whole and the effects of different treatment groups will be analysed using mixed-effects modelling. The data from this study are longitudinal and hence non-standard statistical methods are needed to account for the dependence within subjects. We will use mixed models to account for this dependence. Mixed models allow us to fit a standard regression equation to the fixed effects of time and treatment, and a random effect to differences between participants. This allows us to assess the effects of time and treatment group (and other covariates – baseline vitamin D status, medication adherence) whilst accounting for the longitudinal nature of the data. Two primary treatment groupings will be used as key fixed factors in modelling outcomes:

1. Treatment allocation– outcomes for participants randomised to receive vitamin D will be compared to outcomes for those allocated to placebo;
2. Vitamin D serum concentrations: participants who exhibit an increase in serum concentrations of 25-hydroxy vitamin D will be compared to those not exhibiting an increase in serum concentrations.

We will check the adequacy of the model by examining the residuals for heterogeneity and normality. All analysis will be conducted on an intention to treat basis (i.e. all randomised participants included in the analysis).

6) Give details of the ethical considerations attached to the proposed project:

Use of Vitamin D supplements
We do not expect this protocol will expose the subjects to any appreciable risk. The dose of 25OHD in the active arm is 5000 IU, well below that level that can be obtained after one episode of sunbathing (20,000 IU). There is robust evidence showing that oral supplements in standard doses are safe and that there is a wide margin of safety
